# “Thoroughly out of my depth - A quality improvement project to improve junior clinician’s confidence in undertaking adult ADHD clinic appointments

**DOI:** 10.1192/j.eurpsy.2024.1655

**Published:** 2024-08-27

**Authors:** B. Marron, L. McKeown

**Affiliations:** ^1^NHS Argyll & Bute, Lochgilphead, United Kingdom

## Abstract

**Introduction:**

There has been a 1000% increase in referrals for assessment of adult ADHD within Scotland over the past three years (The Scottish Government. 2023. *NAIT adult Neurodevelopmental Pathways Report).* These referrals are sent by general practitioners to the local community mental health team. The most junior clinicians (doctors who are pre-membership with the Royal College of Psychiatrists) in the team are often responsible for undertaking the initial assessment of these patients. Patients have on average waited almost a year to be seen and expectations are high.

**Objectives:**

The diagnosis of ADHD can be challenging, and adult ADHD is still a relatively new and evolving diagnostic entity. We set out to explore how junior clinicians were coping with this in their daily practice.

**Methods:**

We developed a questionnaire that was sent to all junior clinicians working within Argyll & Bute (n=8) via an anonymised email link. The link was open for 1 week and then results were analysed.

**Results:**

The response rate to our survey was 87.5%. Prior to starting their current roles none of the respondents had ever undertaken an ADHD assessment before. All respondents answered “No” when asked if they felt they had adequate knowledge on ADHD in order to perform assessments. Only 14% (n=1) felt they had access to adequate resources about how to make an ADHD diagnosis. Participants were asked on a scale of 1-10 (1= not at all,10= very) to rate their confidence in conducting ADHD assessments. The average confidence score was 2.43. There was a space for free text feedback in which participants reported the following: “felt thoroughly out my depth”, “I felt chucked in the deep end”, “I felt very under qualified”

**Image:**

**Figure:**
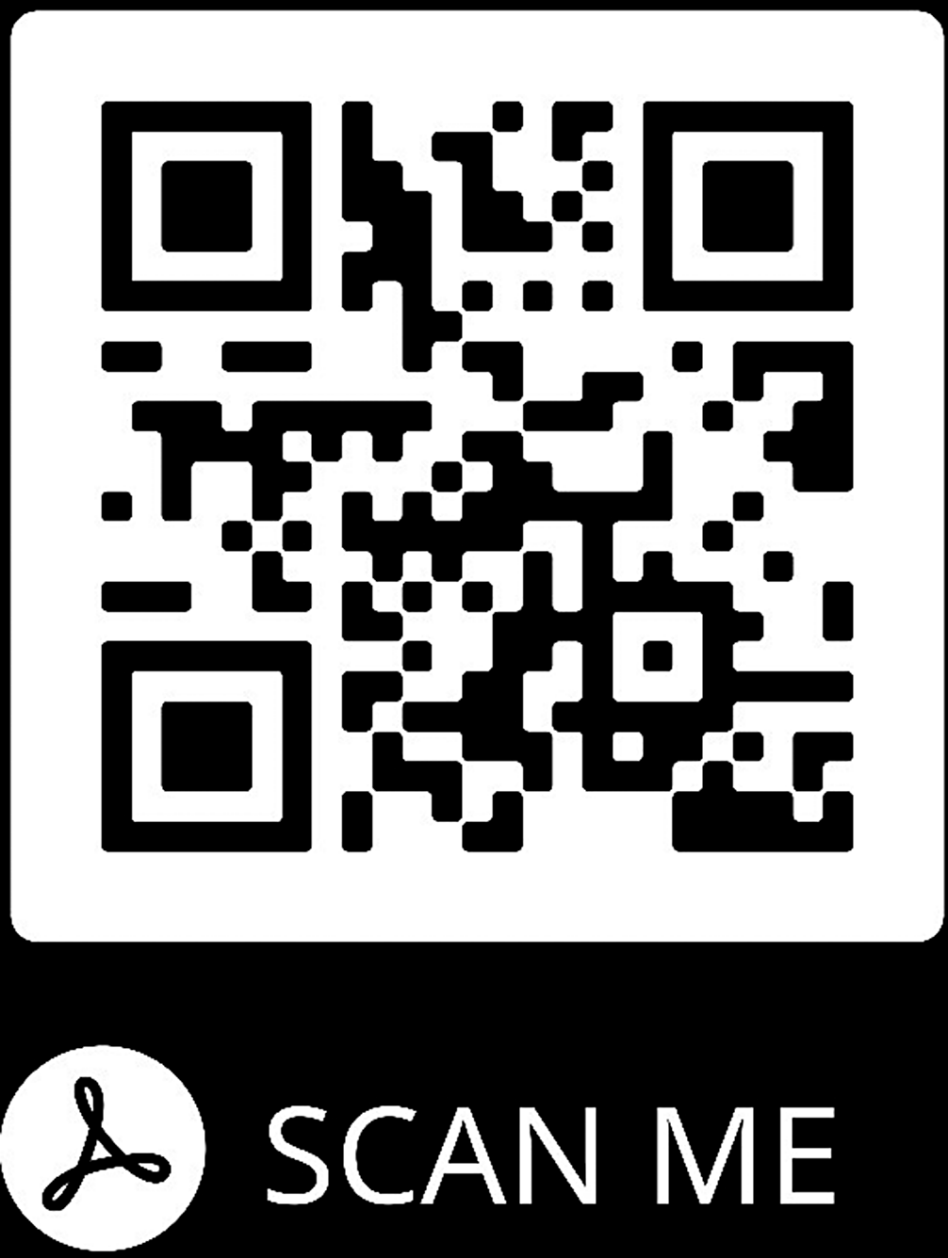

**Conclusions:**

Our results showed that junior clinicians did not feel confident or knowledgeable about undertaking adult ADHD assessments. In response to this we have now produced, in association with the consultant psychiatrists within our health board, an informative and engaging quick reference poster which explains how to undertake an ADHD assessment (see QR code attached as Image 1). It contains useful pointers about diagnosis, and more importantly guidance on language/phrases to use when explaining to patients whether or not you feel a diagnosis of ADHD is appropriate. It then also explains next steps that can be offered i.e. psychological support/medication options and how to prescribe these. We plan to send out a repeat questionnaire to the next cohort of junior clinicians to assess if they have improved confidence in managing and diagnosing ADHD following this intervention.

**Disclosure of Interest:**

None Declared

